# Serum vitamin D is a biomolecular biomarker for proliferative diabetic retinopathy

**DOI:** 10.1186/s40942-019-0181-z

**Published:** 2019-11-05

**Authors:** Gauhar Nadri, Sandeep Saxena, Abbas Ali Mahdi, Apjit Kaur, Md. Kaleem Ahmad, Pragati Garg, Carsten H. Meyer

**Affiliations:** 1 Department of Ophthalmology, King George’s Medical University, Lucknow, U.P 226003 India; 2 Department of Biochemistry, King George’s Medical University, Lucknow, U.P 226003 India; 3 Department of Ophthalmology, Era’s Lucknow Medical College And Hospital, Lucknow, India; 4 Department of Ophthalmology, Pallas Klinik, Aarau, Switzerland

**Keywords:** Vitamin D, Proliferative diabetic retinopathy, Receiver operating characterstics curve, Area under curve

## Abstract

**Background:**

Vitamin D is a multi-functional fat-soluble metabolite essential for a vast number of physiological processes. Non-classical functions are gaining attention because of the close association of vitamin D deficiency with diabetes, and its complications. The present study was undertaken to evaluate the role of vitamin D as a biomarker for proliferative diabetic retinopathy.

**Methods:**

A tertiary care center based cross-sectional study was undertaken. Seventy-two consecutive cases of type 2 diabetes mellitus were included. Diagnosis of diabetes mellitus was made using American Diabetes Association guidelines. Study subjects included: diabetes mellitus with no retinopathy (No DR) (n = 24); non-proliferative diabetic retinopathy (n = 24); and proliferative diabetic retinopathy (n = 24) and healthy controls (n = 24). All of the study subjects underwent complete ophthalmological evaluation. Best Corrected Visual Acuity (BCVA) was measured on the logarithm of the minimum angle of resolution (logMAR) scale. Serum 25-OH Vitamin D assay was done using chemiluminescent microparticle immunoassay technology. Diagnostic accuracy of vitamin D was assessed using receiver operating characteristics curve analysis and area under curve (AUC) was determined for the first time.

**Results:**

ANOVA revealed a significant decrease in serum vitamin D levels with severity of diabetic retinopathy (F = 8.95, *p* < 0.001). LogMAR BCVA was found to increase significantly with the severity of DR (F = 112.64, *p* < 0.001). On AUC analysis, a cut off value of 18.6 ng/mL for Vitamin D was found to be significantly associated with proliferative diabetic retinopathy [sensitivity = 86.36% (95% CI 65.1–96.9); specificity = 81.82% (95% CI 59.7–94.7); AUC = 0.91 (excellent); and Z value = 8.17].

**Conclusions:**

Serum vitamin D levels of ≤ 18.6 ng/mL serve as sensitive and specific indicator for proliferative disease, among patients of DR.

## Introduction

Diabetes mellitus will be the seventh leading cause of death in 2030 [1]. The prevalence of diabetic retinopathy (DR) is closely related to the rise in prevalence of diabetes mellitus [2–5]. A recent pooled analysis from 35 population-based studies projected that 93 million people worldwide have diabetic retinopathy, of whom 17 million (~ 18%) have proliferative DR [6]. Vitamin D is a multifunctional hormone. Its activated metabolite, 1, 25-dihydroxy vitamin D_3_, has diverse biological functions. Vitamin D insufficiency has attained pandemic proportions, with more than half the world’s population at risk [7–9]. Enzymatic conversion (hydroxylation) occurs in the liver and kidney. This is required for activation of the vitamin D pro-hormone to the active form, calcitriol, which exerts its effect via nuclear receptors at several locations [10]. Vitamin D is a crucial regulator of several genes regulating key biological processes. Poor glycemic control increases the risk for the development and progression of DR. In animal models, low vitamin D levels have been shown to impair synthesis and secretion of insulin [11].Therefore, an optimal concentration of vitamin D is essential for efficient insulin secretion and function [12–14]. Also, Vitamin D has anti-inflammatory and anti-angiogenic properties [15–19].

Low serum vitamin D levels have been found to be associated with an increased severity of diabetic retinopathy [20]. We evaluated serum vitamin D as a biomolecular biomarker for proliferative diabetic retinopathy (PDR), for the first time.

## Methods

A tertiary care centre based cross sectional study was undertaken after approval from the institutional review board according to the tenets of the Declaration of Helsinki. Sample size was calculated using 95% confidence interval. An informed voluntary consent was obtained from all the study subjects. Diagnosis of type 2 diabetes mellitus was made according to American Diabetes Association (ADA) guidelines which include fasting plasma glucose level ≥ 126 mg/dl, 2 h plasma glucose level ≥ 200 mg/dl during an oral glucose tolerance test [21]. Seventy-two consecutive cases of diabetes mellitus in the age group of 40–70 years were included. Mean duration of diabetes mellitus, in years, was 7.25 ± 5.63 in No DR, 9.82 ± 5.33 in NPDR, and 10.75 ± 4.63 in PDR groups respectively. Diabetic retinopathy was graded according to the ETDRS classification by two experienced observers masked to the status of diabetic retinopathy of the cases [22]: diabetes patients without retinopathy (n = 24), non-proliferative diabetic retinopathy (n = 24), and proliferative diabetic retinopathy (n = 24). Twenty-four healthy controls were also included. The intergrader agreement was high with a Cohen’s Kappa of 0.85.

None of the study subjects were confined to indoor activity due to poor health and had sufficient outdoor exposure. Patients with any other ocular or systemic disease which could affect the retinal vasculature, systemic diseases like cardiovascular disease, renal failure, diabetic neuropathy and other macrovascular complications of DM, tuberculosis, chronic liver disease, cancer, any prior disease that suggested baseline alterations in vitamin D and calcium metabolism, such as hyperparathyroidism or hypoparathyroidism, or recent nephrolithiasis were excluded. Patients on vitamin supplements, antioxidants, or on any medications causing change in Vitamin D metabolism such as Rifampin, Phenobarbital, and Phenytoin were also excluded. The best-corrected visual acuity (BCVA) was documented on the logMAR scale. All study subjects underwent detailed fundus evaluation using stereoscopic slit lamp biomicroscopy and indirect ophthalmoscopy. Digital fundus photography and fluorescein angiography were done.

Blood samples from study subjects were drawn by aseptic vein puncture and transferred into tubes containing 3.89% trisodium citrate (in the ratio of 9:1) for separation of plasma. Glycated hemoglobin was measured on auto analyzer using standard protocol. Serum 25(OH)D concentration was measured by a chemiluminescence delayed, one-step assay on the Abbott Architect i-1000SR analyser (Abbott Diagnostics, Wiesbaden, Germany).

## Statistics

Data were summarized as Mean ± SE (standard error of the mean). Continuous two independent groups were compared by Student’s t test. Continuous more than 2 independent groups were compared by one way analysis of variance (ANOVA) and the significance of mean difference between the groups was done by Newman-Keuls post hoc test after ascertaining normality by Shapiro–Wilk’s test and homogeneity of variance between groups by Levene’s test. Categorical (discrete) groups were compared by Chi square (χ^2^) test. Pearson correlation analysis was done to assess association between the variables. Independent predictor(s) for severity of diabetic retinopathy was assessed using univariate ordinal logistic regression analysis. Receiver operating characteristics (ROC) curve analysis was done to evaluate the accuracy of vitamin D as a biomolecular biomarker for severity of diabetic retinopathy. Accuracy was measured by the area under the ROC curve (AUC). An area of 1 was considered to represent a perfect test. Traditional academic point system was used as a guide for classifying the accuracy of the diagnostic test: 0.90–1 = excellent; 0.8–0.9 = good and 0.7–0.8 = fair. Two-tailed (*α* = 2) *p* < 0.05 was considered statistically significant. Analysis were performed on SPSS software (Windows version 17.0).

## Results

Table 1 shows Demographic, laboratory parameters and LogMAR visual acuity in Controls, No Diabetic Retinopathy (NO DR), Non Proliferative Diabetic Retinopathy (NPDR), Proliferative Diabetic Retinopathy (PDR).

**Table 1:** Demographic, laboratory parameters and LogMAR visual acuity in controls, no diabetic retinopathy (NO DR), non proliferative diabetic retinopathy (NPDR), proliferative diabetic retinopathy (PDR)

Variables	Controls (n = 22) (%)	NO DR (n = 22) (%)	NPDR (n = 22) (%)	PDR (n = 22) (%)	F/χ^2^ value	*p* value
Age (years)	53.50 ± 1.6	53.24 ± 1.20	53.72 ± 1.40	53.61 ± 1.70	0.03	0.99
Sex						
Female	8 (36.4)	13 (59.0)	6 (27.0)	9 (41.0)	4.90	0.20
Male	14 (64.1)	9 (41.0)	16 (73.0)	13 (59.0)		
Hb (gm/dL)	12.10 ± 0.32	12.0 ± 0.30	11.0 ± 0.50	11.40 ± 0.40	0.84	0.50
HbA1c (%)	5.35 ± 0.11	8.0 ± 0.50	8.20 ± 0.43	8.80 ± 0.60	13.10	< 0.001
Blood sugar F (mg/dL)	83.10 ± 2.10	142 ± 10.12	161.50 ± 11.12	180.90 ± 10.0	23.05	< 0.001
Blood sugar PP (mg/dL)	105 ± 2.00	217.15 ± 15.20	251.0 ± 13.4	261.0 ± 12.28	36.20	< 0.001
Vitamin D (ng/mL)	25.9 ± 1.60	23.30 ± 2.01	18.10 ± 1.90	14.10 ± 1.20	9.05	< 0.001
S. urea (mg/dL)	33.14 ± 0.88	33.26 ± 2.14	44.16 ± 3.44	48.12 ± 2.66	10.42	< 0.01
S. creatinine (mg/dL)	0.71 ± 0.02	0.96 ± 0.05	1.31 ± 0.27	1.57 ± 0.08	4.10	< 0.01
VA (logMAR)	0.10 ± 0.02	0.40 ± 0.04	0.71 ± 0.07	1.2 ± 0.02	113.14	< 0.001

**Fig. 1:**
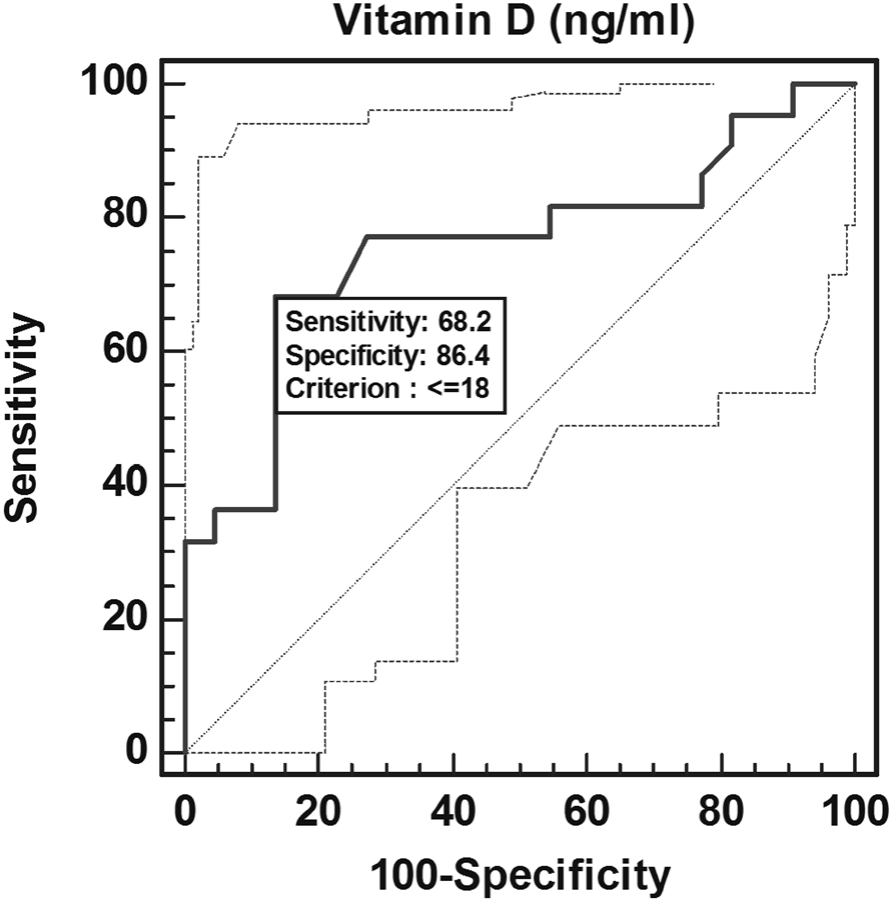
Sensitivity and specificity of serum vitamin D to discriminate controls and cases of non proliferative diabetic retinopathy (NPDR) using ROC curve analysis

**Fig. 2:**
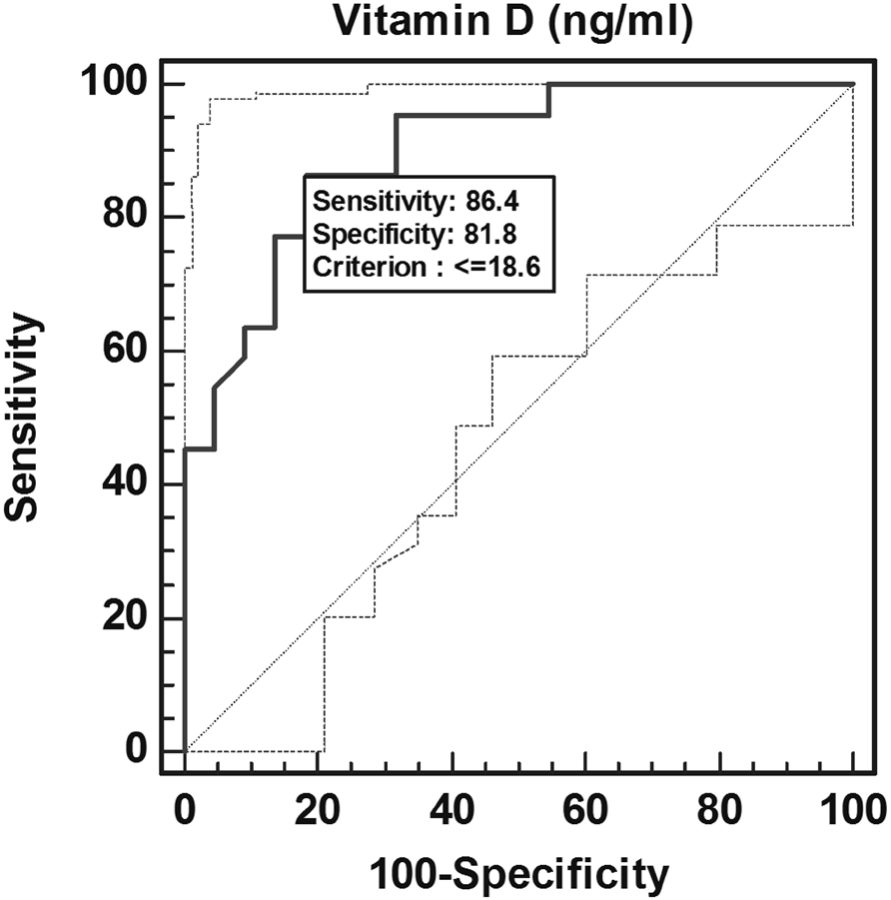
Sensitivity and specificity of serum vitamin D to discriminate controls and cases of proliferative diabetic retinopathy (PDR) using ROC curve analysis

Comparing the mean age and sex of four groups, ANOVA showed similar age among the group (F = 0.03, *p* = 0.9) and χ^2^ test showed similar sex frequency among the groups (χ^2^ = 4.9, *p* = 0.2). Newman–Keuls test showed significantly different and higher values for HbA1c, blood sugar F and PP, VA in cases as compared to controls (*p* < 0.05 or *p* < 0.01 or *p* < 0.001). Univariate ordinal logistic regression analysis found vitamin D as a significant predictor of severity of diabetic retinopathy {OR (95% CI) = 1.11 (1.06–1.16) (*p* < 0.01 or *p* < 0.001)}. ROC curve analysis demonstrated vitamin D cut off value of 18.6 ng/mL to be significantly associated with NPDR and PDR (Table 2, Figs. 1, 2).However, excellent AUC of 0.91with high sensitivity and specificity was observed for PDR.

**Table 2:** Diagnostic accuracy of vitamin D (ng/mL) to discriminate no diabetic retinopathy (NO DR), non proliferative diabetic retinopathy (NPDR), proliferative diabetic retinopathy (PDR) using ROC curve analysis

Group	Sensitivity (95% CI)	Specificity (95% CI)	+ TPV	− PV	AUC	Z value	*p* value
NO DR	40.91 (20.7–63.6)	81.82 (59.7–94.7)	69.2	58.1	0.581	0.93	0.352
NPDR	68.18 (45.1–86.1)	86.36 (65.1–96.9)	83.3	73.1	0.757	3.51	< 0.001
PDR	86.36 (65.1–96.9)	81.82 (59.7–94.7)	82.6	85.7	0.91	8.17	< 0.001

*+* *PV* positive predictive value, *−* *PV* negative predictive value, *AUC* area under the curve

## Discussion

Low vitamin D levels have been found to be associated with increased severity of DR.A recent meta-analysis of fifteen studies involving 17,664 subjects, defined vitamin D deficiency as serum vitamin D levels below 20 ng/mL, and vitamin D insufficiency as serum vitamin D levels of 21–29 ng/mL. This meta-analysis revealed that the subjects with serum vitamin D levels of < 20 ng/mL experienced a significantly increased risk of DR [23]. Another meta-analysis demonstrated that patients with PDR have a statistically significant lower mean serum vitamin D levels than those with NPDR [24].

In the present study, we found low serum vitamin D levels to be associated with PDR (14.10 ± 1.20 ng/mL) and NPDR (18.10 ± 1.90 ng/mL).However higher serum vitamin D levels were observed for NO DR (23.30 ± 2.01 ng/mL) and controls (25.9 ± 1.60 ng/mL). Area under curve analysis, showed that cut off levels of 18.6 ng/mL were significantly associated with occurrence of NPDR and PDR. Excellent AUC of 0.91 for PDR was observed as compared to fair AUC of 0.75 for NPDR. The results indicated that serum vitamin D cut off levels of 18.6 ng/mL were significantly associated with PDR and decrease in serum Vitamin D levels served as a potential biomarker for PDR.

Inflammation and VEGF play a significant role in the pathogenesis of macular edema and neovascularization in PDR. Hypoxia induces VEGF production [25]. Also, oxidative stress and inflammation responsible for RPE dysfunction may lead to abnormal angiogenesis as VEGF is secreted by RPE [26, 27]. Our previous studies highlighted that enhanced oxidative stress, and increased serum VEGF and ICAM-1 levels are associated with an increase in the severity of diabetic retinopathy resulting in an increase in macular thickness and increased grades of RPE alterations [28–32].

Vitamin D has a suppressive role in the pathogenesis of DR via its well recognized anti-angiogenic and anti-inflammatory effects. Mantel et al. in a mouse oxygen-induced ischemic retinopathy model demonstrated that active metabolite of vitamin D, calcitriol, is a potent inhibitor of retinal neovascularization. Vitamin D inhibits VEGF induced endothelial cell sprouting, elongation and endothelial cell proliferation [33]. Also, Albert et al. [34] a mouse model, proposed that vitamin D induces endothelial cell apoptosis, and interrupts the angiogenesis signaling pathway. In human cancer cells, vitamin D has been shown to mediate its anti-angiogenic activity by inhibiting the transcription of hypoxia-inducible factor (HIF-1) [15]. Chronic Inflammation results in protein damage, aggregation and degeneration of RPE. Vitamin D exerts an anti-inflammatory effect by inhibiting the proliferation of natural killer cells, lymphocytes and several pro inflammatory cytokines. Vitamin D also inhibits the production of the metalloproteinase, MMP-9, released by inflammatory cells [35].

Limitations of the present study are small sample size and cross sectional design, as causality cannot be determined. In conclusion, this study shows that patients with PDR, had lower vitamin D levels.

AUC suggests vitamin D as a simple, sensitive and specific, laboratory investigative indicator for proliferative diabetic retinopathy, among cases of DR. Studies with larger sample size are suggested for further evaluation.

## Data Availability

The datasets used and analysed during the current study are available from the corresponding author on reasonable request.

## Abbreviations

DR: diabetic retinopathy
NPDR: non proliferative diabetic retinopathy
PDR: proliferative diabetic retinopathy
ROC: receiver operating characterstics
AUC: area under curve
RPE: retinal pigment epithelium
VEGF: vascular endothelial growth factor
